# Rural reality contradicts the ethnographic literature—a nationwide survey on folk beliefs and people's affection for the stork in Poland

**DOI:** 10.1186/s13002-024-00689-6

**Published:** 2024-05-14

**Authors:** Andrzej Wuczyński, Agnieszka Pieńczak, Gabriela Krogulec

**Affiliations:** 1 Institute of Nature Conservation, Polish Academy of Sciences, A. Mickiewicza 33, 31-120 Kraków, Poland; 2 Institute of Culture Studies, Faculty of Humanities, University of Silesia, Uniwersytecka 4, 40-007 Katowice, Poland; 3 Stowarzyszenie Rzeczniczki Przyrody (Nature’s Advocates Association), Walecznych 7/11, 50-341 Wrocław, Poland

**Keywords:** Ethnobiology, Cultural heritage, Biodiversity conservation, Birds, *Ciconia ciconia*, Culture and religion studies, Central Europe

## Abstract

**Background:**

Due to early synanthropization and ecological and behavioural features, the White Stork *Ciconia ciconia* became the most cherished of European birds. Rooted in human culture, the species has been well studied; nevertheless, knowledge of people’s attitudes and stork-related folk beliefs remain descriptive. Here, we attempt to quantify these issues in the world's largest stronghold of the species, Poland, in the 1950s.

**Methods:**

The study is based on recently discovered, original nationwide survey data from the 1958 International White Stork Census. These materials were gathered to assess the population size, but they also included issues belonging to the humanities. We have worked them up in a quantitative manner, which has resulted in an original approach rarely found in ethnological studies. We aim to propose an original typology of stork-related beliefs, their spread and regional diversity in Poland and the relationship with stork abundance.

**Results:**

A sample of 2343 questionnaires revealed that affection towards storks was widespread (91.4% positive responses), more so in eastern Poland. The most frequent beliefs relate to respect for the stork (65%) and prophesies (24%), thereafter parental beliefs (7%) and stork biology (3%). Positive attitudes and the dissemination of beliefs increased with stork densities but were unrelated to the respondents’ sex. Utilitarian beliefs outweighed those prioritized in ethnographic studies (e.g. the stork’s human origins) or popular today (baby-bringing), and expressed the real concerns of country folk.

**Conclusions:**

The discovery of long-lost data bordering on ethnology and nature conservation and their novel work-up highlights a realistic dimension of the human–nature relationship and provides a benchmark for further interdisciplinary research.

**Supplementary Information:**

The online version contains supplementary material available at 10.1186/s13002-024-00689-6.

## Introduction

Human beings have interacted with animals since time immemorial, and their culture is replete with references to them. Those contacts usually resulted from their utility or because of the risks they posed; but human-animal relationships go beyond simple utilitarian considerations [1]. There have also been supernatural associations, illustrated in a plethora of fairy tales, folk beliefs and rites, shaping the lives of human communities, people’s attitudes towards animals and affecting animal populations themselves [2, 3]. Traces of old beliefs and customs, though often seemingly incomprehensible, have survived to modern times. Getting to know them makes it possible to gain greater insight into the human–nature relationship, its importance in the moulding of folk culture and in the impact of humans on the environment. Although this relationship is nowadays no longer so strong [4], the human and natural domains are still connected and embedded in webs of interactions with implications for the sustainable management of natural resources [5].

The White Stork *Ciconia ciconia* (henceforth: stork) is an excellent subject for studying human–nature relationships. It is one of the best-researched species of wild birds, a classical model in population studies [6, 7]; it is also an umbrella and indicator species widely used to stimulate conservation awareness [8]. At the same time, thanks to the long history of coexistence with humans and the many links with cultural practices, the stork, the most cherished of European birds, is considered a “social-ecological keystone species” [9] or a “culturally important species” [10]. People’s affection for the stork is expressed, for instance, by its being recognized as a national symbol (in Alsace, Denmark, Lithuania, Poland), in reintroduction programmes [11] and assistance in erecting nest platforms or overwintering sick birds, practices that have been in existence for centuries [12], or the popularity of nest webcams [13]. But although it is widespread, this affection is hard to quantify.

Storks have been present in folk rituals and beliefs since ancient times [14]. But it was not until the second half of the nineteenth century, when ethnographic and ethnological studies were gathering pace, that the importance of storks in folklore became a focus of scientific interest. Revealed were the great variety of myths, beliefs, proverbs and folk tales associated with the stork, such as the motif of the human origin of these birds, the ban on killing them or their role in weather forecasting and matrimonial predictions, to name but a few examples [15, 16]. The development of ethnographic cartography in the 1930s enabled source materials relating to traditional cultures to be gathered [17], and ethnographic atlases were published in many countries [18]. Some of these works brought to light stork-related folk themes from present-day Poland, western Belarus and Ukraine [15, 19], Germany, Austria and Czechia [20–22]. It was remarked upon in passing that the prevalence of stork-related beliefs differed from region to region. These are very widespread in Slavic lands, where the stork’s distribution range is centred, where the stork is revered as an exceptional bird, intimately associated with humans, benevolent and sacred [23–26]. In Poland, the conviction that storks have a human origin is more commonly held in the north-east of the country than in the west [15, 27]. The origins of these regional differences are hard to discern. Are they the result of the differing rates at which new cultural phenomena spread [24, 28]? Has it something to do with the tempo of agricultural development, e.g. differences in rural affluence? Or do they perhaps derive from features of stork populations, such as their abundance or the numbers of nests constructed on buildings?

Many ethnographic works contain detailed descriptions of these beliefs and their variants, but in the context of the present paper, it is precisely this descriptive character that is troublesome. The quantitative approach is missing, otherwise one could estimate the relative importance of specific beliefs. What is also surprising is the lack of a coherent system of beliefs that is independent of geographical or temporal frameworks. True, these beliefs are discussed thematically, e.g. a symbol of childbirth or divination of the future, but they are grouped arbitrarily, even in excessive detail. This hampers cognition of their real meaning and their prevalence, and hence the opportunity to carry out interethnic comparisons.

Poland is ideally suited to assess the stork’s place in traditional culture because the country lies in the centre of the bird’s distribution range and has a rich cultural heritage. With its large area (312,679 km^2^), diverse habitats and regions of extensively managed farmland, Poland is of global importance for the White Stork, hosting the world's largest population, assessed at 30,500 (1984)–52,700 (2014) breeding pairs [29]. The relationship between storks and humans is reinforced by the fact that these birds build their nests almost exclusively among human habitations; indeed, in areas with the highest stork densities, there may be several tens of them in one village.

Poland can also boast a long and continuing tradition of stork research [12]. A recent global review has shown that the highest number of peer-reviewed publications on storks during the last 70 years has come from Poland [30]. The stork continues to be an important feature of the Polish cultural heritage: it has been present in Polish traditional culture for many centuries [12, 31–33]. Nowadays, it is treated as a symbol of the country and its farming landscape, and very often figures in all manner of marketing and promotional campaigns for urban and regional development, not to mention tourism and rewilding projects [9, 34].

This paper summarizes the extensive material obtained from a mid-twentieth century, nationwide questionnaire on people’s attitudes to the stork and stork-related beliefs. This information was gathered in passing during a country-level census of White Stork nests [35, 36]. But the questionnaires were never analysed, the information they contained on stork-related beliefs was forgotten, and no similar study was ever undertaken again. Their discovery after 60 years revealed a vast store of data that was highly original, among other things because they were collected incidentally during ornithological, not ethnographic research. We have attempted to analyse these data using the typical approach in biological research, focusing on the quantitative, not the qualitative. The aims of the study were as follows: (i) to produce a quantitative description of people’s attitudes to storks; (ii) to create a universal typology of beliefs applicable to other areas inhabited by the White Stork (from the wide range of the questionnaire and the variety of beliefs described, it is very likely that they cover most of those existing in Europe); (iii) to state the frequency of the particular types of belief in Poland (to assess which of them are of real significance to people); (iv) to search for regional differences in the frequency of specific types of belief (anticipated in light of Poland’s large area and its cultural diversity, and also differences in stork abundances; (v) to evaluate the effect of selected variables contained in the questionnaires on the prevalence of these beliefs.

## Materials and methods

### Data source

The analysis is based on long-lost survey data, collected nationwide during the 2nd International White Stork Census (1958) [36]. At that time, the Polish stork population numbered 46,100 breeding pairs, the largest in the world, and has remained at roughly the same level to this day [35]. The nationwide density was 14.7 pairs/100 km^2^ but was uneven, increasing along the SW-NE axis. The survey data also included issues relating to the humanities, i.e. people's attitudes and beliefs concerning the stork (hereafter ABS), and these issues are examined in this work.

The questionnaires were addressed to the smallest administrative units (“gromada”), covering several villages, the aim being to reach the largest number of the c. 40,000 villages existing in Poland in 1958. The survey forms were sent out to 8,339 gromadas (94.9% of the gromadas in Poland) and a relatively high return of 73.6% (6,139 gromadas) was obtained [37]. The actual recipients of the questionnaires were teachers in rural schools, who were asked to provide information on the occurrence of storks in the immediate vicinity of the school, based on their own knowledge and interviews with pupils.

The questionnaires (Additional file 1: Fig. S1) contained two questions relating to ABS: 4. “People’s attitudes to storks (are they regarded as useful or harmful, why? are they persecuted or protected, why?), and 5. “Are there any superstitions relating to storks? If so, what are they?” The following aspects make these specific questions, and also the 1958 questionnaires as a whole, unique: (i) the nationwide reach of this survey, a rarity in ethnographic studies (an exception was the research for the Polish ethnographic atlas (see [27]); (ii) the fact that the materials were gathered during routine counts of storks, addressing issues from a fascinating mix of ornithology, conservation biology and ethnology; (iii) these data were obtained within 15 years of the end of World War II, when folk beliefs were still quite strongly held in rural Poland and were regionally very diverse, while at the same time, atlases of folk culture and other works were being published, which we used as comparative material (see “Introduction” section); (iv) the questions relating to folk beliefs encroached into the realm of spiritual culture, more difficult to grasp in ethnographic surveys than social or material culture; (v) the questions relating to beliefs were open, the intention being to acquire spontaneous responses (no doubt the respondents told of the beliefs that they thought were the most important or the most widespread); this distinguishes this questionnaire from categorized interviews conducted in accordance with a pre-prepared survey form [38] (Additional file 2: Fig. S2), an aspect that is highly relevant to the present work; (vi) as it turns out, this questionnaire is also unique because no subsequent stork survey (count) ever contained questions about ABS, so comparative analyses are not possible.

### Questionnaire sampling and classification

**Fig. 1:**
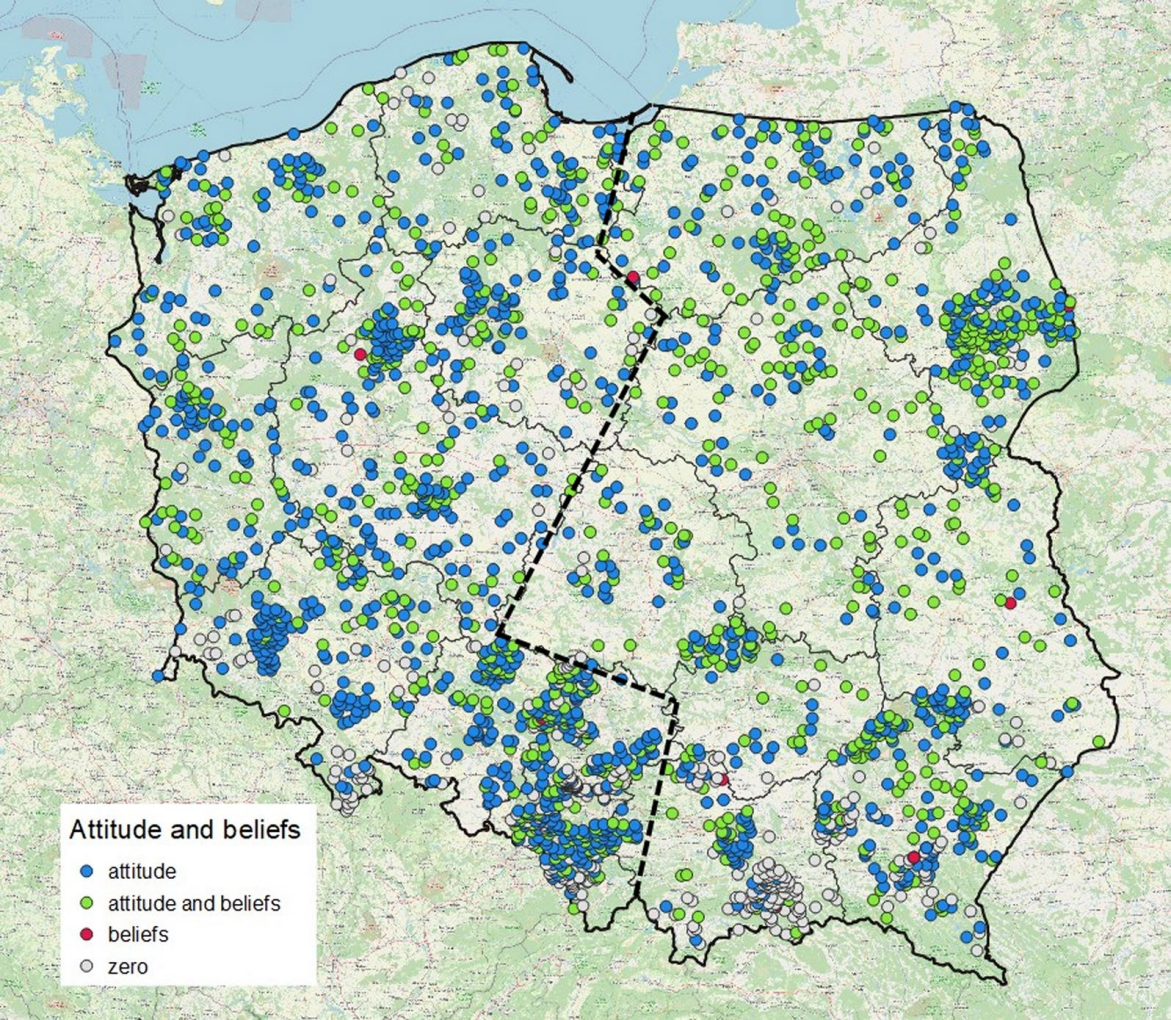
Spatial distribution of the 2343 questionnaires sampled from the 1958 survey describing people's attitudes towards storks and folk beliefs relating to these birds (some questionnaires were left blank), on the map of Poland divided into provinces. The broken line roughly divides the provinces of eastern and western Poland, used in the statistics (see text)

The analysis is based on a sample of 2343 questionnaires (38.2% of those returned): some describe people's attitudes (1783, 76.1%) and beliefs (756, 32.3%) towards storks, others make no mention of these aspects (538, 23.0%). The questionnaires were chosen at random within the main administrative units in Poland, provinces and districts (Fig. 1), in order to achieve nationwide coverage. The survey is incomplete only in areas from which no questionnaires are available. In order to take a quantitative approach to people’s attitudes to storks, we distinguished four attitude categories: positive, negative, mixed and neutral (Table 1), which were usually declared unequivocally in the questionnaire. The ‘mixed’ category was invoked wherever the stated positive or negative causes were ambiguous. The declared attitudes were motivated by various causes, defined as utilitarian, aesthetic, associated with respect for the stork or its inconvenience.

**Table 1:** Classification of people’s attitudes towards storks, including the reasons for the attitudes and examples taken from the questionnaires

Attitude towards storks	Reason for the attitude The causes are defined as respect/reverence for the stork (R), utilitarian (U), aesthetic (E), inconvenience (I)	Example	Location
Positive	- Belief in the beneficial features of storks (they bring luck, protect against fire, diseases, evil, predators) (R) - they eat “vermin” (animals perceived as harmful, e.g. rodents, or obnoxious, e.g. reptiles (U) - Aesthetic reasons (they adorn the farm and the countryside; they traditionally accompany the farmer) (E) - The prestige attached to having a stork’s nest on one’s property (R)	“People consider that storks are useful and don’t persecute but protect them, saying that the stork is a second farmer, which monitors, as it were, the freshly ploughed land, feeding on beetle larvae and other small animals.”	Świdry, d. Giżycko, p. Olsztyn
		“People don’t do them any harm; they regard a stork’s nest as adorning a building. People collect storks’ feathers for disinfecting children.”	Klonowa Góra, d. Suwałki, p. Białystok
Negative	- They consume domestic animals (chickens, goslings, fish fry, young hares) (U) - Fear of storks (they are vengeful, bring bad luck, carry adders onto farms) (R) - The stork is a troublesome neighbour (it damages roofs, sullies the farmyard) (I)	“People think that storks destroy thatched roofs; there was even one case where one of their nests was deliberately destroyed.”	Mirotki, d. Starogard Gdański, p. Gdańsk
		“They are generally regarded as harmful; every year they take chickens or ducklings, but we protect them because tradition demands this of us.”	Sumina, d. Rybnik, p. Katowice
Mixed	Various combinations of the above reasons	“Most people protect storks. But there are some who claim that a stork brings bad luck and so destroy any nests close to houses.”	Brzozowa, d. Sulęcin, p. Zielona Góra
		“Some people believe that storks are useful, but without justifying such a belief. Most people, however, think that storks are pests because they feed on all kinds of birds’ eggs, chicks/nestlings, they consume young hares and bees in the meadows, etc. They are protected as the first harbingers of spring, so nobody does them any harm.”	Markotów, d. Kluczbork, p. Opole
Neutral	- A neutral attitude towards nature - Rational treatment of storks	“The local populace generally takes a neutral stance towards storks. Only one farmer took pains to erect a nest for the storks. These birds are not persecuted, but neither are they regarded as harmful.”	Sieroty, d. Gliwice, p. Katowice
		“People are generally indifferent to storks. They are neither persecuted nor taken care of. They are left to fend for themselves.”	Wolica Brzozowa, d. Tomaszów Lubelski, p. Lublin

The names of the town, district (d.) and province (p.) from which the questionnaire originates are given in the last column

In order to classify stork-related beliefs, we first reviewed the European literature, noting those functioning in both Polish and foreign folklore. Then we perused some 500 questionnaires to compare the range of beliefs from both sources. It turned out that although there were many variations in the details, the fundamental features of the beliefs recurred again and again, e.g. relating to the harvest in a given year based on eggs or nestlings discarded from the nest, how many of them, and so on. But always the basis was the fate of clutches/broods, or more generally, storks allowed people to foretell the future. Similarly, the presence of a nest in the farm was a blessing, gave protection against fire, or testified to the farmer’s good qualities, which generally led to beliefs expressing respect for the stork. From this review we were able to draw up a classification of beliefs applicable to the various regions where storks occur (Table 2). We distinguished four main categories, i.e. beliefs relating to respect or reverence for the stork, prophesies, childbirth and stork behaviour, and a further eight sub-categories. The last category—stork behaviour—does not contain any sub-categories because of the small number of responses and their quite broad differentiation, which would hamper classification. It is also worth highlighting the fact that this is the only category focused on the stork itself; the others focus on people, with the stork being treated in utilitarian fashion.

**Table 2:** Classification of stork-related folk beliefs, including the basis of these beliefs and examples taken from the questionnaires

Major categories	Detailed categories (beliefs related to:)	Variants and the basis of beliefs	Example beliefs	Location
Reverencing beliefs	Fire	- Protection against fire - Storks cause fires	“Where there is a stork’s nest no lightning strikes.”	Zawyki, d. Łapy, p. Białystok
			“It used to be said that if you harmed a stork, it might set the building on fire. It would do this by clapping its bill so vigorously that sparks would fly, thereby starting the fire that would consume the building.”	Ostrynka, d. Sokółka, p. Białystok
	Happiness	- Bringing good luck - Protection of the farmhouse and village - The stork was originally a human being or an angel - The stork is sacred - A stork’s nest offers testimony of a good farmer	“The stork brings happiness and good fortune to a family. The houses on which storks have built their nests are happy homes”	Majdan Zbydniowski, d. Tarnobrzeg, p. Rzeszów
			“It is said that storks build their nests on the houses belonging to good people.”	Szarów, d. Bochnia, p. Kraków
			“A stork is a man punished by God for his inquisitiveness. God gave him a sack full of serpents, but instead of throwing it into the abyss, the man opened it to see what was inside. The snakes wriggled free and crawled off in all directions, so as punishment he has been gathering them up to this day.”	Żarków, d. Krosno Odrzańskie, p. Zielona Góra
	Revenge	Revenge (but not as causing a fire) for killing or injuring storks, be they adult birds, young ones, their eggs or nests	“Whosoever kills a stork, his wife will go crazy, his cow will not calve, and his children will fall ill.”	Solnica, d. Nowy Dwór Gdański, p. Gdańsk
			“If a stork is killed, blood rain will fall.”	Suków, d. Kielce, p. Kielce
Prophetic beliefs	Weather and harvest	Prophesies based on: - A stork’s behaviour (flying or standing), - A stork’s appearance (clean/dirty) - The fate of the clutch/brood and the nest (eggs or nestlings thrown out of the nest) - Foraging area	“If a stork brings couch grass into the nest, that means it’s going to rain.”	Dąbie, d. Koło, p. Poznań
			“People generally believe that if a stork throws an egg out of the nest, it’s going to be a good year with a bumper harvest, but if it throws a nestling out, the year is going to be difficult and the harvest a bad one.”	Łubianka, d. Koło, p. Poznań
	Disasters		“If storks abandon their nests, there’s bound to be a fire.”	Kiełpiniec, d. Sokołów Podlaski, p. Warsaw
			“If storks throw their eggs out and abandon their nests, ill fortune will befall the whole village.”	Jeżów, d. Brzeziny, p. Łódź
	Family and health		“If you see the first stork to arrive back in spring in flight, you will have a happy year or you will go on a journey (move house). If that stork is on the ground, you won’t be going anywhere, life will go on as usual. But if you see that stork standing on one leg, someone in your family is going to die.”	Boguszyn, d. Jarocin, p. Poznań
			„People believe that if a stork nests on an inhabited house, someone in that family is going to die.”	Żernica, d. Gliwice, p. Katowice
Parental beliefs	Bringing babies	Prophesies based on: - Stork behaviour - Numbers of storks (adults, nestlings)	“Grown-ups explain to their children that storks catch babies in the water and bring them home”	Folwarki Wielkie, d. Białystok, p. Białystok
			“They bring the babies down the chimney and though the windows.”	Szczecinowo, d. Ełk, p. Białystok
	Matrimonial		“If storks are having a fight, the young miss living in that farmhouse will have all the boys running after her."	Dobryszyce, d. Radomsko, p. Łódź
			“Unmarried girls wish to see an even number of flying storks—that means they will soon be wedded.”	Cieszacin Mały, d. Jarosław, p. Rzeszów
Stork behaviour	The behaviour of storks in relation to their mates, other storks, other animals		“Before flying off for the winter, the storks assemble in a ‘parliament’. They test their flying abilities, clap their bills. Weaklings are discarded. They choose a leader.”	Ugoszcz., d. Białogard, p. Koszalin
			“If nails are driven into a harrow or a frame, storks won’t settle there because the nails will attract lightning.”	Czechowice, d. Bielsko-Biała, p. Katowice

### Database and analyses

The classified ABS were entered on to a common spreadsheet (MS Excel), in which the basic unit was the questionnaire from a particular school (village). We also took zero questionnaires into account, that is, those not containing any mention of ABS; even so, these did enable us to assess how widespread stork-related beliefs were. If several different beliefs were given in a particular questionnaire, each was entered separately on to the spreadsheet; this meant that the total number of beliefs in a particular category was greater than the overall number of nonzero records. Each record was georeferenced corresponding to the town or village where the school was located; the results could thus be presented graphically. Apart from this visualization, the regionalization of ABS was checked by dividing Poland along administrative borders into an eastern part (8 provinces, 167 999 km^2^) and a western part (9 provinces, 144 680 km^2^) (Fig. 1). This division reflects the differences in the intensity of farming in Poland (greater in the west), accessible key habitats of the stork [39] and its abundance [29]. In order to find links between selected ABS drivers, attributed to each record were the number of stork nests in the surveyed village and the sex of the teacher who filled out the questionnaire. We anticipated that larger numbers of storks would coincide with more strongly held beliefs, but might also limit positive attitudes to storks [40, 41]. We also expected that men would take a more neutral attitude towards storks than women, and would tend to hold beliefs relating to their farmwork, e.g. those affecting harvests, whereas women would be more likely to hold beliefs associated with spirituality and children. Because the distributions of variables were skewed, the differences were analysed using nonparametric tests. The significance of differences relating to frequency were chi^2^-tested. Differences in stork abundance in association with ABS were examined using the Kolmogorov–Smirnov test because of the high number of linked ranks, that is, villages with the same number of nests. The association between the prevalence of beliefs with stork numbers was tested only in the sample of villages with a minimum of one nest because of the possible demotivating effect of the absence of storks on the respondent.

## Results

### People’s attitudes towards storks

**Fig. 2:**
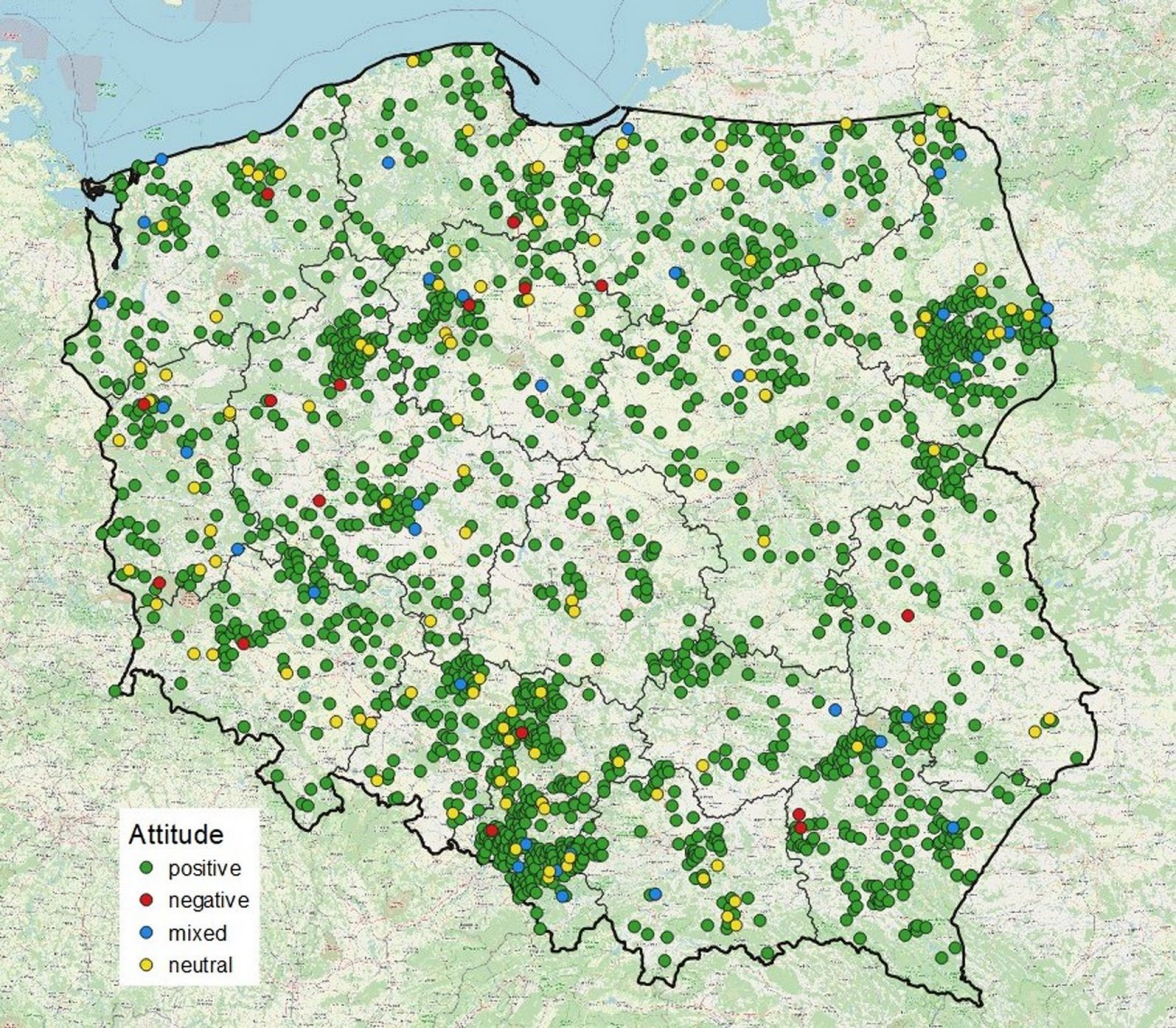
Distribution of questionnaire responses expressing different types of attitudes towards storks in Poland (*N* = 1783)

The questionnaires revealed the overwhelmingly positive attitude towards storks in Poland, declared one hundred times more often than a negative one (Table 3). The causes of positive attitudes were predominantly utilitarian—in most cases, it was that storks consume animals regarded as pests—but the negative attitude was engendered by their eating domesticated animals. Positive attitudes were quite often (36%) aesthetically motivated. Positive attitudes were prevalent throughout Poland, but very strongly so in the east of the country, whereas negative and neutral attitudes were more common in the west (Fig. 2, Additional file 3: Table S1).

**Table 3:** People’s attitudes to the stork in Poland in 1958 and the reasons for positive and negative attitudes expressed quantitatively

People’s attitudes	Number of questionnaires	Percentage of questionnaires
Positive	1630	91.4
Neutral	102	5.7
Mixed	35	2.0
Negative	16	0.9
Total	1783	100.0
Reasons for positive attitudes		
U—utilitarian	185	48.2
E—aesthetic	138	35.9
R—associated with respect/reverence	61	15.9
Total	384	100.0
Reasons for negative attitudes		
U—utilitarian	22	56.4
I—associated with inconvenience	14	35.9
R—associated with respect (fear)	3	7.7
Total	39	100.0

### Stork-related beliefs

**Fig. 3:**
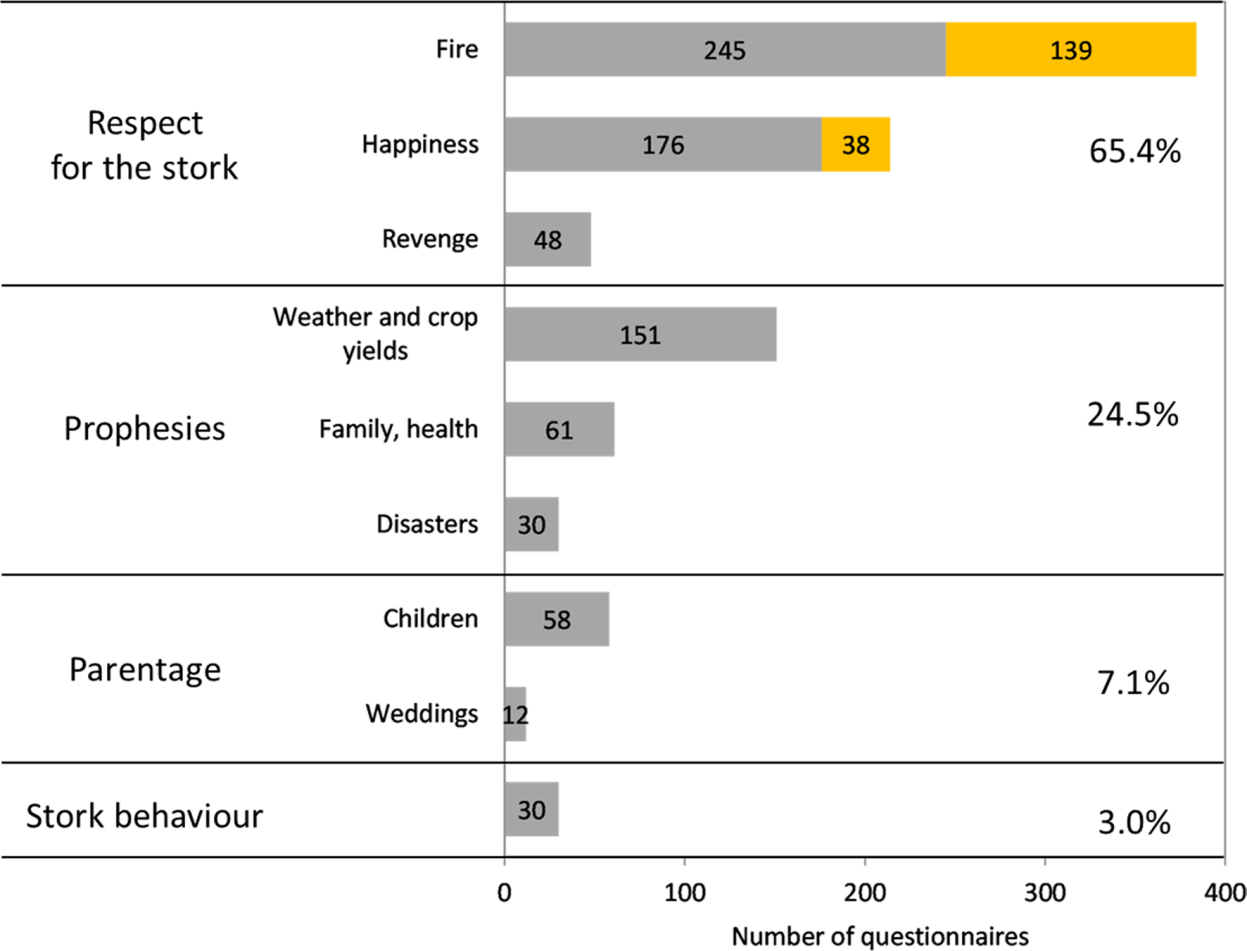
Frequency of stork-related beliefs in light of a nationwide survey carried out in 1958. The belief categories are as in Table 2. The values on the bars denote the numbers of responses referring to a particular type of belief (*N* = 988). The Fire bar has been split into responses relating to protection against fire (grey) and those concerning storks causing fires (yellow). Again, the Happiness bar has been divided into a grey area (storks bring good fortune) and a yellow one (all other aspects, i.e. blessing for the farmhouse, the stork’s origins in humans or angels, testimony of the good husbandman)

**Fig. 4:**
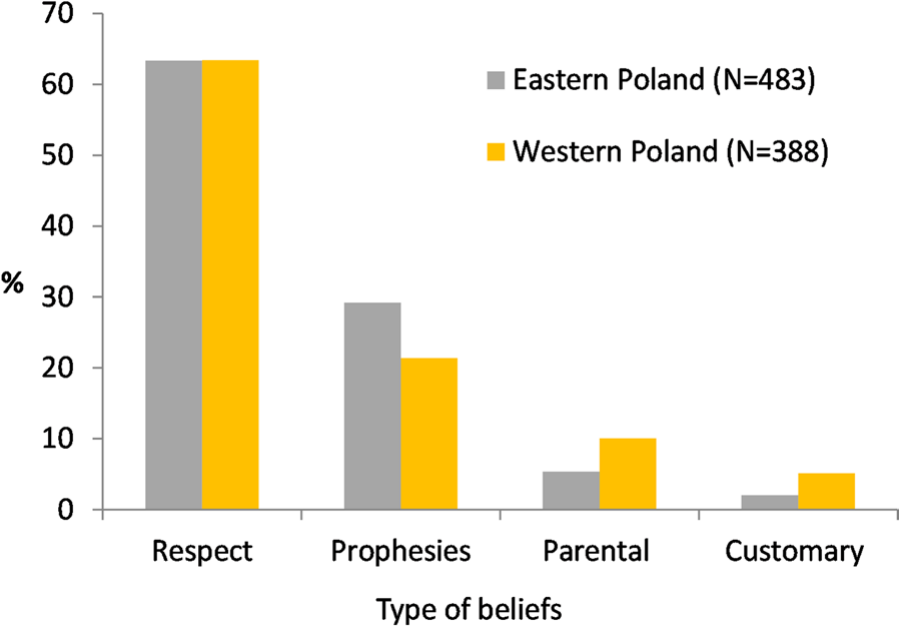
Frequency of the main categories of stork-related beliefs in eastern and western Poland (chi^2^ = 17.32, df = 3, *P* = .001)

**Fig. 5:**
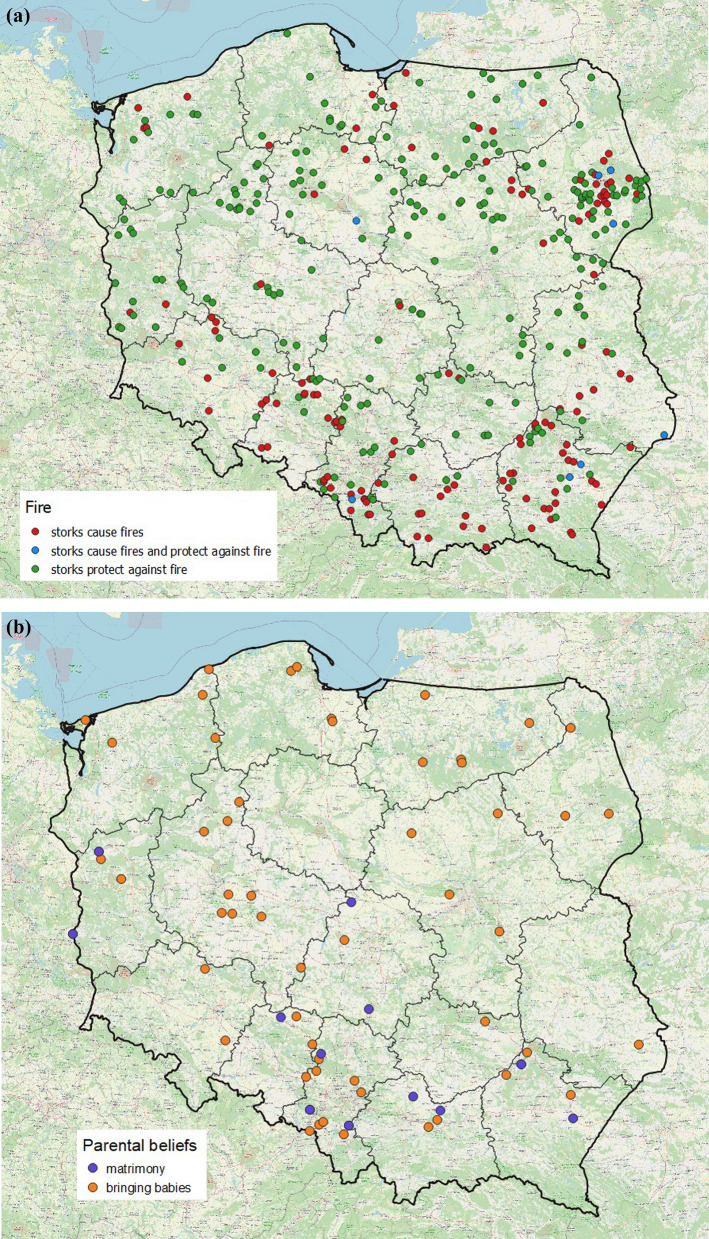
Distribution of beliefs referring to **A** protection of farmhouses against fire or storks causing fires (*N* = 384) and **B** storks bringing babies and matrimonial prophesies (*N* = 70)

The frequency distribution of stork-related beliefs in the above categories was uneven (Fig. 3). As many as 90% of the responses referred to respect and prophesies, and the other 10% to childbirth and stork behaviour. The most widespread were beliefs associated with fire (39% in all), whereby protection against fire (conflagration, lightning strike) was distinctly more common than fires being caused by storks. Also frequent was the conviction that storks bring good fortune, but there were only sporadic mentions of other aspects associated with luck/happiness: for example, the myth that storks take their origins from humans occurred just six times. Prophetic beliefs mostly concerned the weather and crop yields. Very surprising was the quite small percentage of parental beliefs, especially the still very popular baby-bringing myth. Beliefs referring to the behaviour of storks made only occasional appearances, among which care for their young, relations with other birds and the avoidance of metallic objects in their nests were recurring themes. The percentage of prophetic beliefs was higher in eastern Poland, whereas in the west, beliefs about childbirth and stork behaviour were more prominent (Fig. 4). The percentages of beliefs relating to protection against fire or storks causing fires did not differ between the eastern and western parts of the country (chi^2^ = 1.36, df = 1, *P* = 0.243, *N* = 384), but differences tended to be distributed latitudinally. The myth about storks causing fires was especially prevalent in south-eastern Poland (Fig. 5a). Among the parental beliefs, the baby-bringing myth was present throughout Poland, but matrimonial beliefs occurred almost exclusively in the south (Fig. 5b).

### Drivers of people’s attitudes and beliefs

One thousand four hundred thirty questionnaires stated the sex of the teachers: 516 women and 914 men. Sex was not linked to ABS—both men and women revealed very much the same frequencies of attitudes (chi^2^ = 0.04, df = 2, *P* = 0.979, *N* = 1176) and beliefs (chi^2^ = 0.83, df = 3, *P* = 0.842, *N* = 583) towards storks, corresponding to general values.

There were from 0 to 22 stork nests in the villages analysed here (av. = 2.36, *N* = 2335) and the number of nests was significantly correlated with ABS (Table 4). There were more stork nests in villages where attitudes towards storks were positive than in those places where attitudes were negative or neutral. Moreover, there were more storks’ nests in villages from which beliefs of any type were described than from where no beliefs were described.

**Table 4:** The average number of stork nests in villages with respect to the declared attitude towards storks and the presence or absence of beliefs

ABS	Av. no. of nests ± SD	N	Av. no. of nests ± SD	*N*	*d* (Kolmogorov–Smirnov test)	*p*
Attitude	Positive		Negative or neutral			
	2.99 ± 2.89	1625	2.25 ± 2.47	118	0.132	< .05
Belief	Present		Absent			
	3.65 ± 3.07	704	2.95 ± 2.69	995	0.124	< .001

Because negative responses were few in number, questionnaires with negative and neutral attitudes were pooled. Questionnaires with mixed attitudes were not taken into account

## Discussion

Based on an archival dataset from the 1958 White Stork census in Poland, we discuss, for the first time, people’s attitudes and beliefs related to storks in this large European country, situated in the centre of the species’ geographical range. The 1958 questionnaire was criticised for its excessive detail, a factor that could have been responsible for the ultimate fiasco of that year’s count [42]. As a result, all subsequent questionnaires were simplified and focused solely on questions regarding the abundance of storks. We show, however, that the detailed nature of the 1958 questionnaire, and especially its secondary aspects, revealed a wealth of information from several disciplines, which was never again obtained on such a scale, either in Poland or anywhere else within the stork’s distribution range. Moreover, when navigating this borderland between population ecology, nature conservation and ethnology, we were taking a wholly new approach to ethnographic data. We decided not to go into the minutiae of the beliefs described, as the variations could have been due to the respondents’ fertile imagination or their messages being misinterpreted. Instead, we invoked a universal typology of beliefs and attempted to quantify them in order to highlight those belief themes that were really relevant in rural Poland. These assumptions yielded a realistic picture of the human–nature relationship, with the result that this system can probably be applied anywhere in the stork’s distribution range and in different human cultures.

**Fig. 6:**
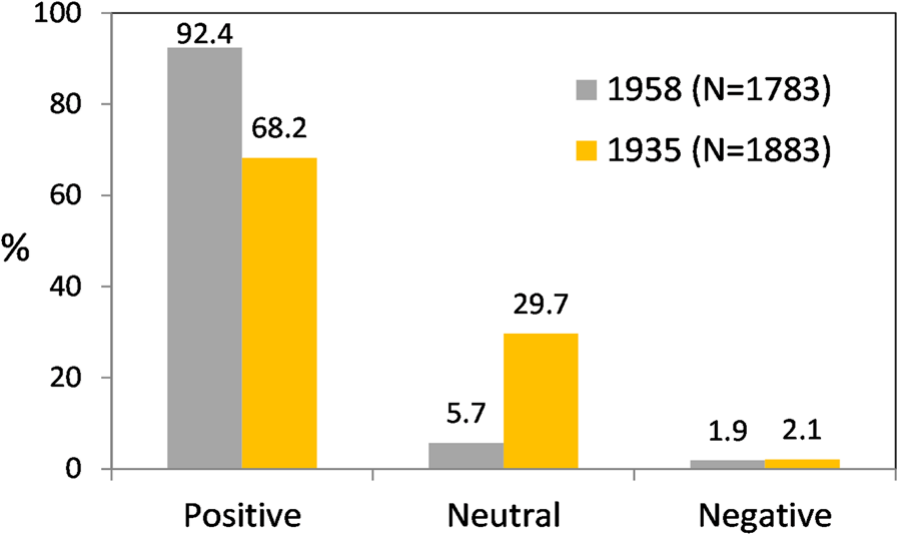
Comparison of attitudes towards storks in light of the nationwide survey of 1958 (in the present paper, mixed attitudes have been pooled with positive and negative ones) and from the 1930s, in three provinces of southern Poland (chi^2^ = 358.7, df = 2, *P* < .001)

Our data confirm the universal affection for the stork in Poland in the mid-twentieth century. Positive attitudes towards storks were a hundred times more common than negative ones, and only 1% of respondents regarded storks as problematic. This result is hardly surprising, especially in light of the multifarious stork conservation projects in Poland and elsewhere in Europe [13], https://www.whitestorkproject.org. Formerly, however, it was far from obvious. The only comparative quantitative data available from Poland come from three pre-war provinces in the south of the country (Silesia, Kraków and Lwów (Lviv)), an area of 47 708 km^2^), where in 1933–35 a similar questionnaire survey was carried out [43–45]. There, too, attitudes towards storks were generally positive, though at a lower level than in the 1958 survey; far more frequent were neutral opinions, and locally there were cases where these birds were deliberately eradicated (Fig. 6). Wodzicki [43] deemed such a situation to be transitory, however, emerging as it did from unjustified suspicions that storks were responsible for substantial losses among game animals, as reported in the late-nineteenth-century German hunting literature. Our data from 1958 indicate a much warmer relationship with the stork, probably resulting from a greater environmental awareness, the introduction of legal protection for animals and the restricted access to firearms after World War II.

It is not surprising either that utilitarian reasons for the affection or antipathy towards storks were dominant, that is, because they fed on animals, respectively, regarded as pests or useful ones. It is interesting, however, that these attitudes were often held for aesthetic reasons or as a result of superstitions. The latter arguments were put forward by people who, though ecologically illiterate, had very intimate practical relationships with nature. This confirms the existence of an aesthetic and spiritual affinity for nature among country folk. Such convictions were soon re-expressed in more formalized concepts of philosophical anthropology, like eco-aesthetics, biophilia or ecosophy [46, 47], but in the mid-twentieth century there were no empirical data available to support them. Most certainly, they stem from belief traditions more remote in time [48]. In nineteenth century landed estate records we read that country folk who mistreated a stork were employed on estates only with great reluctance, while in regions where stork densities were high, villages and farms not inhabited by storks were ostracized by their neighbours, for examples, when marriages were being arranged [13]. This exemplifies the positive effect of beliefs on the practical protection of storks, in the same way as the effect of traditional, negative myths regarding the eradication of wolves, ravens, owls or snakes [49, 50].

The questionnaires confirmed the diversity of beliefs widely held in rural Poland. All the types of belief, known from the anthropological and ethnographic literature, came to light, along with a multitude of variants which have served to enrich our knowledge of such beliefs. We obtained our new results by classifying them quantitatively. Interestingly, the beliefs were predominantly treated in an anthropocentric manner. As many as 97% of the beliefs related to three types—respect/reverence, prophesies and childbirth—which de facto are focused on human beings. The remaining 3% were related to the last type, i.e. stork behaviour, which were focused on these birds themselves. This implies that people protected storks, respected and/or revered them or even feared them, were happy to use them for making prophesies, but only secondarily bothered with impractical beliefs that describe the stork’s biology or its legendary human origins.

More specifically, the most widespread beliefs had to do with fire (lightning strikes). The stork was meant to protect people and their possessions from fire, or less commonly, it was said to cause fires. The predominance of this myth was no doubt due to the frequency with which fires used to break out in rural areas, a fact that is rarely remarked upon, urban conflagrations having then been far more spectacular (https://en.wikipedia.org/wiki/List_of_town_and_city_fires). With the crude means of heating rural houses and cottages and the inflammable materials, like thatch, used to build them, fires in country areas used to be a real hazard. The large dimensions of storks’ nests, exposed on the roofs of buildings, could have significantly increased the risk of a lightning strike; indeed, medieval codices of urban law prescribed the removal of storks’ nests from the roofs of tenement houses [51]. This is confirmed by contemporary figures on the causes of stork mortality in Poland: 3.1% of nestlings and 1.3% of fully-fledged birds perished as a result of lightning strikes [52]. Observations have also shown that lightning striking a stork’s nest does not always kill the birds, which will have further encouraged the development of myths and superstitions (Additional file 4: Appendix S1). The stork’s red legs and bill were identified with the causation of fire [16], whereas specific observations of stork parents sprinkling their nestlings with water during very hot weather [53] were associated with the farmhouse being protected against fire.

The sources of some prophetic beliefs are also to be found in actual observations of storks. The connection between the weather and the phenology of migration and breeding success has been empirically confirmed in a great many population studies of the White Stork [54]. This can be correlated with the analogous influence of the weather on crop yields in a given year, a frequent belief motif. Besides these realistic themes, there are also fantastic beliefs, relating to vengeance on farmers, the stork’s ability to foretell disasters or the fates of families being tied to the behaviour of the first storks seen in spring.

The greatest surprise was the low frequency of what is nowadays the commonest stork-related belief, that these birds bring babies. Although it has existed in different European cultures for a very long time [55, 56], this belief only became widespread in the nineteenth century, reaching the peak of popularity in the late twentieth century, no doubt as a result of its presence in advertising, pop-culture and media development. By contrast, the stork as a symbol of childbirth was mentioned a mere 58 times in the more than 2300 questionnaires from the 1958 survey. Perhaps this state of affairs is due to the ornithological nature of that survey and the open-endedness of the questions. By comparison, this belief was recorded as many as 126 times on 272 questionnaires filled out for the purposes of the Polish Ethnographic Atlas, these data having been gathered mainly in the 1980s (source: PEA archives).

We found no relationship between the sex of a teacher respondent and the responses relating to ABS. This may have been due to the credibility of the teachers, who objectively summarized what they could remember in this respect. Some of their responses were very likely based on interviews with their pupils, which obviously made the final result independent of the sex of the person signing the questionnaire. It is interesting to note that the proportions of men and women teachers signing the questionnaires were not equal: men did so twice as often as women. This would also suggest that in the Poland of those days it was men who formed the core body of teachers, in contrast to the present-day situation.

On the other hand, the results were evidently dependent on the abundance of storks. Beliefs were stated significantly more often in villages with more nests, which would suggest that the close proximity of storks and their constant contacts with people fostered their creation and dissemination. Less pronounced was the link between the numbers of storks and peoples’ attitudes. We had anticipated that larger numbers of storks might cause them to be regarded as troublesome neighbours [41]. Quite the contrary, in fact: larger numbers of storks did not lessen people’s affection for these birds; indeed, the more nests in a village, the more often were attitudes positive. Probably, the typical situation in Poland (from one to a few nests per village) was that stork numbers were below those that would make these birds irksome. Interestingly, positive attitudes were expressed more often in eastern Poland, which used to be regarded as economically backward and whose inhabitants did not include non-indigenous people. This stands in contrast to the more wealthy western parts of Poland, where a fair proportion of the populace were immigrants [39, 57]. This confirms the intimate relations that sedentary societies had with nature and throws light on the possible weakening of these relationships as these traditional communities became wealthier [4, 58]. In the case of the stork, relevant data will be needed to verify this.

Summarizing, the major results of this analysis include the documentation of the universal affection for the stork in the Poland of 1958, as well as the drawing up of the first taxonomy of stork-related beliefs and their quantification. A specific achievement is the revelation of forgotten questionnaires from the mid-twentieth century and the extraction of ethnographic data from ornithological materials. The ethnographic theme in them necessarily consisted of open-ended questions, which was actually an advantage. The information acquired revealed what people really thought about the stork in rural Poland. It turned out that beliefs, such as the one about storks being derived from humans, prettily embellished as tales in ethnographic works, were by no means widespread. Neither were beliefs then common that are popular nowadays, like the myth of storks bringing babies, which appeared quite late in the anthropological literature [59]. In fact, a highly practical rural reality emerged from these questionnaires. Villagers recognized the beauty of the stork, expressed the traditional association with this bird and protected it, but their stork-related beliefs contained mainly information that was useful to them in their everyday lives. In validating the above, the extensive empirical data from the centre of the stork’s distribution, analysed in this paper, may serve as a point of reference for similar studies in other parts of the stork’s range. The temporal aspect is also important: here we have shown the state of affairs prevailing in the mid-twentieth century. How these beliefs have subsequently evolved is an intriguing question. Their characteristics and frequency in contemporary rural Poland and Europe ought to be the subject of a new questionnaire or of similar surveys being carried out at the present time.

### Supplementary Information

**Additional file 1**. Figure S1. A sample survey form on the occurrence of the White Stork in Poland, used during the International White Stork Census in 1958.

**Additional file 2**. Figure S2. Questions relating to animals, applied in research into folk knowledge and beliefs for the 1980s Polish Ethnographic Atlas.

**Additional file 3**. Table S1. People’s attitudes to the stork in eastern (N=837) and western Poland (N=946). The figures denote the percentage of questionnaires reflecting a particular attitude (chi^2^ =6.55, df=2, P=.038).

**Additional file 4**. Appendix S1. A film showing lightning striking a White Stork’s nest, Wola Żytowska, Province of Łódź, Poland, 10.04.2018.

## Data Availability

The questionnaires of the 2nd International White Stork Census (1958) that support the findings of this study are not openly available. The questionnaires are located in controlled access data storage in the Institute of Nature Conservation, Polish Academy of Sciences, Kraków, Poland.

## Abbreviations

ABS: People's attitudes and beliefs concerning the white stork
PEA: Polish Ethnographic Atlas
