# Supplementary material for: Rural reality contradicts the ethnographic literature—a nationwide survey on folk beliefs and people's affection for the stork in Poland
Source: J Ethnobiol Ethnomed. 2024 May 14;20:51. doi: 10.1186/s13002-024-00689-6 (PMC11094895; doi:10.1186/s13002-024-00689-6)

**Rural reality contradicts the ethnographic literature – a nationwide survey on folk beliefs and people's affection for the stork in Poland**

Andrzej Wuczyński, Agnieszka Pieńczak, Gabriela Krogulec

**Figure S1**

A sample survey form on the occurrence of the White Stork in Poland, used during the International White Stork Census in 1958 (Skoroszów, a village in the district of Trzebnica, province of Wrocław)


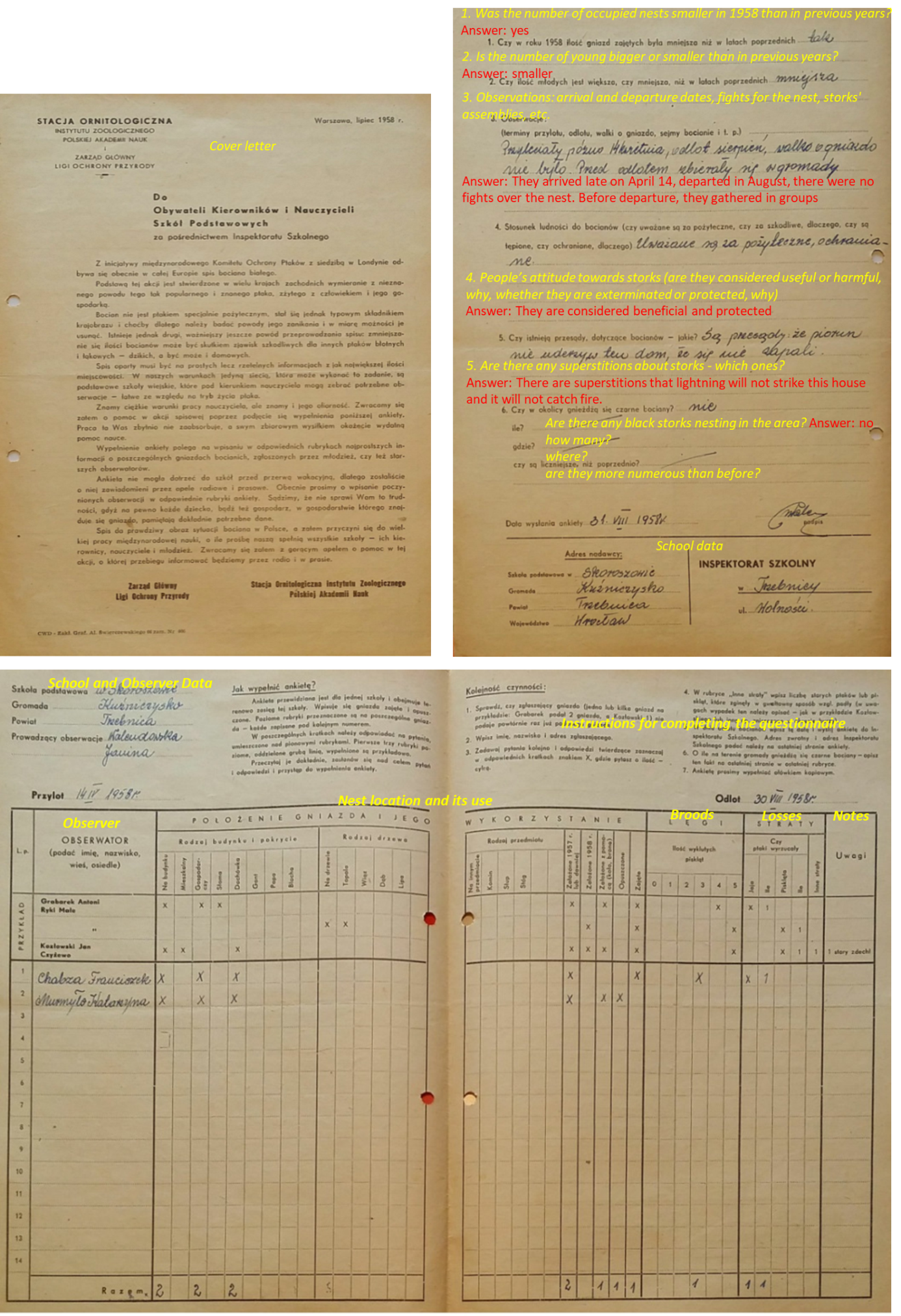

Supplement: Supplementary file 1 — Additional file 1. Figure S1. A sample survey form on the occurrence of the White Stork in Poland, used during the International White Stork Census in 1958. [file 13002_2024_689_MOESM1_ESM.docx]
