# Supplementary material for: Rural reality contradicts the ethnographic literature—a nationwide survey on folk beliefs and people's affection for the stork in Poland
Source: J Ethnobiol Ethnomed. 2024 May 14;20:51. doi: 10.1186/s13002-024-00689-6 (PMC11094895; doi:10.1186/s13002-024-00689-6)
Supplement: Supplementary file 2 — Additional file 2. Figure S2. Questions relating to animals, applied in research into folk knowledge and beliefs for the 1980s Polish Ethnographic Atlas. [file 13002_2024_689_MOESM2_ESM.docx]

**Rural reality contradicts the ethnographic literature – a nationwide survey on folk beliefs and people's affection for the stork in Poland**

Andrzej Wuczyński, Agnieszka Pieńczak, Gabriela Krogulec

**Figure S2**

Questions relating to animals, applied in research into folk knowledge and beliefs for the 1980s Polish Ethnographic Atlas (Lebeda 2002; Kłodnicki et al. 2017). The questions relating to the stork are highlighted (box). Notice that most of the questions are closed (categorized) and only the first one is open: *What do people say about the stork?*

**
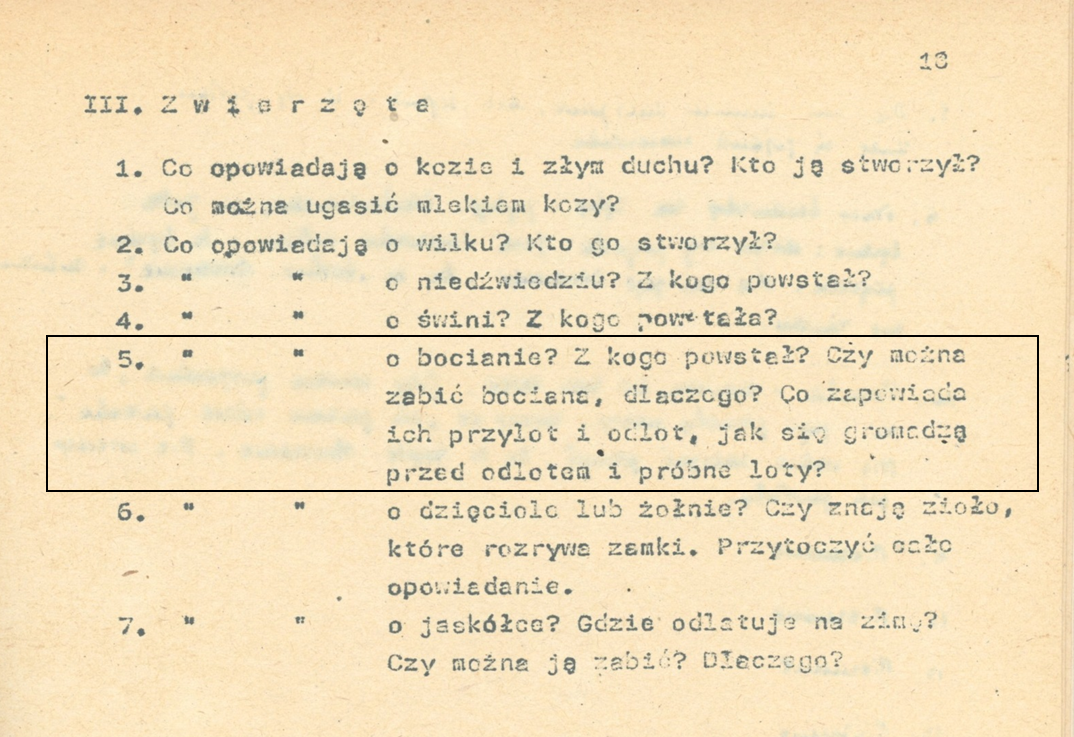
**

1. What do people say about the goat and the evil spirit? Who created them? What can be quenched with goat’s milk?
2. What do they say about the wolf? Who created it?
3. What do they say about the bear? From whom did it originate?
4. What do they say about the pig? From whom did it originate?
5. What do they say about the stork? From whom did it originate? Is it permitted to kill a stork, why? What do their arrival and departure foretell, how do they assemble before departure and trial flights?
6. What do they say about the woodpecker or bee-eater? Do they know any herbs that can open locks? Quote the whole story.
7. What do they say about the swallow? Where does it fly to for the winter? Is one allowed to kill it? Why?
