# Supplementary material for: Rural reality contradicts the ethnographic literature—a nationwide survey on folk beliefs and people's affection for the stork in Poland
Source: J Ethnobiol Ethnomed. 2024 May 14;20:51. doi: 10.1186/s13002-024-00689-6 (PMC11094895; doi:10.1186/s13002-024-00689-6)
Supplement: Supplementary file 3 — Additional file 3. Table S1. People’s attitudes to the stork in eastern (N=837) and western Poland (N=946). The figures denote the percentage of questionnaires reflecting a particular attitude (chi2 =6.55, df=2, P=.038). [file 13002_2024_689_MOESM3_ESM.docx]

**Rural reality contradicts the ethnographic literature – a nationwide survey on folk beliefs and people's affection for the stork in Poland**

Andrzej Wuczyński, Agnieszka Pieńczak, Gabriela Krogulec

**Table S1**

People’s attitudes to the stork in eastern (N=837) and western Poland (N=946). The figures denote the percentage of questionnaires reflecting a particular attitude (chi^2^ =6.55, df=2, *P*=.038).

| People’s attitudes | Eastern Poland | Western Poland | Total |
| --- | --- | --- | --- |
| Positive | 93.0 | 90.1 | 91.4 |
| Negative or neutral | 5.0 | 8.0 | 6.6 |
| Mixed | 2.0 | 1.9 | 2.0 |
| Total | 100.0 | 100.0 | 100.0 |
